# Body Pain Intensity and Interference in Adults (45–53 Years Old): A Cross-Sectional Survey in Chongqing, China

**DOI:** 10.3390/ijerph13090887

**Published:** 2016-09-07

**Authors:** Xianglong Xu, Bing Li, Lingli Liu, Yong Zhao

**Affiliations:** 1 School of Public Health and Management, Chongqing Medical University, Chongqing 400016, China; xianglong1989@126.com (X.X.); 13110265315@163.com (L.L.); 2 Research Center for Medicine and Social Development, Chongqing Medical University, Chongqing 400016, China; 3 The Innovation Center for Social Risk Governance in Health, Chongqing Medical University, Chongqing 400016, China; 4 School of the Second Clinical, Chongqing Medical University, Chongqing 400016, China; chongyilb@sina.cn

**Keywords:** body pain intensity, body pain interference, adults, middle-aged, China

## Abstract

Culture and national care models matter both in reporting and treatment of pain status. However, most findings on body pain intensity and interference in adults are from Western studies, with little reliable evidence from China. This study aimed to assess body pain intensity and interference and its associations with demographic, socioeconomic characteristics, and health behaviors in adults. A cross-sectional survey was performed to collect data from 1224 adults, who were recruited via multistage stratified random sampling. The SF-36 quality-of-life instrument was used to investigate body pain intensity and interference. Ordinal logistic regression analysis was used in this study. Our results showed that 64.1% of the participants (males: 687; females: 537) reported body pain, and 45.7% of the participants reported body pain interference. Middle-aged respondents who were female, were unmarried/divorced or separated/widowed, had a negative relationship with their family, had poor sleep quality, and were not satisfied with their current living conditions had a higher body pain intensity rating (ordered logistic regression/six-level pain intensity criterion; odds ratios, *p* < 0.05). Respondents who were unmarried/divorced or separated/widowed, with a low education level, were unemployed, had lower incomes, had a negative relationship with their family, and were not satisfied with their current living conditions had a higher body pain interference rating (ordered logistic regression/five-level pain interference criterion; odds ratios, *p* < 0.05). In conclusion, an estimated 64.1% of middle-aged adults reported body pain, and 45.7% of middle-aged adults reported body pain interference. These results provide a clue for possible interventions for improving body pain intensity and interference in adults, especially among middle-aged people. These factors should be taken into consideration in the prevention of pain, pain management and treatment planning in order to help relieve the stress of pain among adults.

## 1. Introduction

Pain is an unpleasant and emotional subjective feeling [1]. Body pain intensity evaluates the perceived amount of pain experienced during the previous four weeks. Pain is highly prevalent globally. The World Health Organization (WHO) indicated that 20% of the global population experiences various degrees of chronic pain [2]. People in developing countries may be more likely to suffer from chronic pain than those in developed countries [3]; a more advanced economy corresponds to lower reported incidence of chronic diseases (24.8% vs. 28.1%, respectively) [4]. In the USA, 11.2% of the adult population, which accounted for 35% of adults aged 45–64 years, experienced chronic pain [5]. Pain reporting and treatment rates (6.2% and 28.3%, respectively) were lower in China than those in other countries (≥14.3% and 35.8%, respectively) [6]. Body pain has an influence on normal life and the ability of people to work [7]. The annual government cost in the USA for treating 100 million adults with chronic pain is $560–$635 billion [8]. Approximately 13% of the working population tends to suffer from pain within two weeks, thereby reducing work capacity and productivity, which in turn causes approximately $61 billion of annual losses in the USA [9]. Patients with chronic pain commonly experience cognitive decline [10] and negative emotions [11]. The increased spread of pain is also associated with the prevalence of some cardiovascular diseases [12].

Factors related to pain and interference has been reported elsewhere. People with higher stress tend to have higher pain intensity and interference [13]. Females (aged 40–64 years) reported greater rates of chronic widespread pain than their male counterparts [14]. People aged 70–75 years have the lowest prevalence of chronic pain, whereas people aged 65–70 years old have the highest prevalence of chronic pain [15]. Marriage was associated with pain-related symptoms [16]. Perople with pain in emerging countries (particularly China) are reported to have a higher socioeconomic status trend than those with pain in developed countries [6]. Within the middle age group of 40–64 years, people had a trend of increasing rates of chronic pain with a lower socioeconomic position in the United States [14]. The incidence of chronic pain in rural areas is lower than that in urban areas in China [17]. Pain interference is associated with age, gender, financial status, experiences, education, employment, poor health and sleep, marital status, and high pain intensity [18,19,20,21,22,23]. And higher pain interference is also associated with greater psychopathology and poorer psychiatric treatment response [24,25,26].

Numerous studies have documented rates and risk factors of body pain intensity and interference; however, most findings are from Western studies, with little reliable evidence from China. Little is known about risk factors of body pain intensity and interference among Chinese adults. Clarifying the factors that affect body pain intensity and interference is necessary to pain prevention, management and treatment planning in China. Culture and national care models matter to both reporting and treatment of pain status [27]. The prevalence of pain was higher among African-Americans than among non-Hispanic white people [28]. A previous study showed that 2.2% of adults suffer from pain that lasts at least one day during the past six months, and 25.8% experience pain that lasts at least three months in Chongqing, China [29]. In view of these results, this study aimed to evaluate body pain intensity and interference in adulthood and their associations with demographic, socioeconomic characteristics, and lifestyle factors. In this study, body pain intensity was used to evaluate the perceived amount of pain experienced during the previous four weeks. Body pain interference was used to evaluate the extent to which pain interfered with normal work activities.

## 2. Materials and Methods

### 2.1. Study Design and Participants

Detailed descriptions of the study design and methods, including the population sample, the sampling framework, the survey administration, and the pilot study have been previously reported [30]. A three-level stratified random sampling was used for the sampling procedure, which is designed to choose eligible people with known probability. At stage 1, 10 districts and counties were randomly selected from 38 districts and counties in Chongqing. At stage 2, listing eligible villages were involved within the selected districts and counties. In each selected district/county, approximately 8 to 10 villages were selected (stage 2). Participants were randomly selected from each household in each village (stage 3). Those who agreed were interviewed face to face by investigators to answer every designed question. Within each village, participants were randomly selected to conform to the requirements of the study during July 2009. A total number of 1250 participants were included in the survey. The response rate of these participants was 1230 (98.4%), and six responses were disregarded because of missing data. Finally, 1224 participants were included in the analysis.

### 2.2. Survey Method 

A questionnaire survey was employed to determine if people were born in the specified situation of nutritional health and quality of life. The questionnaire was divided into two sections. The first section determined the general basic information of the participants, including their gender (categorized as male and female). Age was categorized as 51–53, 48–50, and 45–47 years old. Education level was classified as basic education (less than or equal to a primary and junior middle school education level), secondary education (more than or equal to a senior high school, including vocational/technical secondary school and junior college education level), and higher education (more than or equal to a senior college and university education level). Job conditions were categorized as unemployment, employed, and stay at home. Marital status was recorded as married and unmarried/divorced/widowed. The average monthly income was categorized as <¥850 ($124), ¥851 ($124) to ¥1,600 ($234), and >¥1,601 ($234). Regular physical activity was categorized as seldom, sometimes, and usually. Regular daily activities were categorized as seldom, sometimes, and usually. Self-reported sleep quality was categorized as good, average, and poor. Family relationships were categorized as harmonious, average, and poor. Relationships with colleague or friends were categorized as harmonious, average, and poor. Current living conditions were categorized as satisfactory, average, and not satisfied.

### 2.3. Ethics

This study was approved by the Ethics Committee of the Chongqing Medical University (2014013). All participants submitted written informed consent.

### 2.4. Pain Measure

Pain was primarily measured using body pain intensity (e.g., “How much body pain have you experienced over the past four weeks?” (1 = none to 6 = extremely severe)), and body pain interference ((“During the past four weeks, how much has pain interfered with your normal work, including your work in and outside your home?”) (1 = not at all to 5 = extremely)), and items described in the SF-36 quality-of-life instrument [31]. Both questions were structured based on how they were answered.

### 2.5. Statistical Analyses

The characteristics of the participants were summarized using frequencies/percentages and were then presented via descriptive analysis (percentages). Ordinal logistic regression analysis was conducted to examine the risk factors of body pain intensity and interference among participants. Factors were considered while modeling the predicting factors that affect body pain intensity and interference; these factors include age, gender, education level, job conditions, marital status, average monthly income, regular physical activity, regular daily activities, sleep quality, relationship with family, and current living conditions. The statistical tests contained a two-sided test with the statistical significance set to *p* < 0.05. All data analyses were performed using statistical software (SAS version 9.1; SAS Institute, Cary, NC, USA).

## 3. Results

### 3.1. Characteristics of the Sample

Of the 1224 participants, 687 (56.1%) were males. 64.1% of the total number of the participants reported body pain and 45.7% reported interference. Moreover, 28.8% suffered from very slight pain intensity, 25.5% suffered from slight pain intensity, 7.7% suffered from average pain intensity, 1.9% suffered from severe pain intensity, and 0.3% suffered from extremely severe pain intensity. Of the 1224 participants, 34.6% have little pain interference, 9.9% have moderate pain interference, 1.1% has severe pain interference, and 0.2% has extremely severe pain interference. Secondary education was achieved by 49.8% of the 1224 participants, and 34.9% obtained higher education. Of the 1224 participants, 44.0% were unemployed and 45.5% were employed. 22.3% of the total number of participants have a monthly income of less than ¥850, 42.8% have a monthly income of more than ¥1,601, and 34.9% have a monthly income of ¥850–¥1,600. Of the 1224 participants, 23.0% experienced regular physical activity and 6.5% experienced regular daily life. A total of 40% of the participants have good sleep quality. Finally, of the 1224 participants, 75.7% had good relationships with their family, and 52.7% were satisfied with their current living conditions (Table 1).

### 3.2. Ordered Multivariate Logistic Regression Analysis of Body Pain Intensity

Model 1 was adjusted for gender, education level, job conditions, marital status, average monthly income, regular physical activity, regular daily activities, sleep quality, relationship with family, and current living conditions. Model 2 was adjusted for age, gender, education level, job conditions, marital status, average monthly income, regular physical activity, regular daily activities, sleep quality, relationship with family, and current living conditions. Age was not significantly associated with higher body pain intensity rating. Males were less likely to have a higher body pain intensity rating than females (95% confidence interval (CI) (−0.539, −0.106)). The participants who were married or cohabitation were less likely to have a higher body pain intensity rating than the participants who were unmarried/divorced or separated/widowed (95% CI (−0.864, −0.079)). The participants who had good sleep quality were less likely to have higher body pain intensity than those who had poor sleep quality (95% CI (−0.886, −0.072)). The participants who had harmonious (95% CI (−1.887, −0.295)) relationships with their family were less likely to have a higher body pain intensity rating than those who did not get along with their family. The participants who were satisfied with their current living conditions (95% CI (−1.558, −0.518)), or who had an average satisfaction with current living conditions (95% CI (−1.174, −0.170)) were less likely to have a higher body pain intensity rating than those who were not satisfied with their current living conditions. However, age was not significantly associated with body pain intensity in adulthood (Table 2).

### 3.3. Ordered Multivariate Logistic Regression Analysis of Body Pain Interference

Model 1 was adjusted for gender, education level, job conditions, marital status, average monthly income, regular physical activity, regular daily activities, sleep quality, relationship with family, and current living conditions. Model 2 was adjusted for age, gender, education level, job conditions, marital status, average monthly income, regular physical activity, regular daily activities, sleep quality, relationship with family, and current living conditions. Compared with the participants who were 45–47 years old, people who were 48–50 and 51–53 years old were not significantly associated with having a higher pain interference rating. Compared with people who had basic education, people who had secondary education (95% CI (−0.983, −0.328)) and higher education (95% CI (−1.251, −0.439)) were less likely to tend to have a higher pain interference rating. In contrast to unemployed people, employed people were less likely to have a higher trend of pain interference rating (95% CI (−0.868, −0.352)). People who were married or cohabitation were less likely to have a higher trend of pain interference rating than people were unmarried/divorced or separated/widowed (95% CI (−0.945, −0.086)). In contrast to people with an average monthly income below ¥850, people whose average monthly income was ¥851–1,600 (95% CI (−1.048, −0.398)) or above ¥1,601 (95% CI (−1.138, −0.401)) were less likely to have a higher pain interference rating. Compared with people with poor relationships with their family, people who had a harmonious relationship with their family were less likely to have a higher pain interference rating (95% CI (−1.703, −0.038)). Compared with those who were not satisfied with their current living conditions, people who were satisfied (95% CI (−1.821, −0.741)) or averagely satisfied (95% CI (−1.438, −0.400)) with their current living conditions were less likely to have a higher pain interference rating (Table 3).

## 4. Discussion

This study showed that 64.1% of the participants reported body pain, and 45.7% of the participants reported body pain interference. A previous study conducted in Chongqing showed that 25.8% experienced pain that lasted for at least three months [29]. Of the adults, 10.97% reported chronic pain that caused substantial or severe impairment, thereby underscoring persistent pain as a significant societal problem that may affect the daily lives of millions of Chongqing residents [29]. This finding indicates that middle-aged adults may have a higher rate of body pain and body pain interference. A survey conducted in Southwest China showed that 37.9% of hospitalized patients had persistent chronic pain [32]. Middle-aged adults reported a higher rate of body pain than those in Southwest China. Thus, paying attention to body pain among middle-aged adults in Chongqing is necessary.

This study found the relationship between some demographic factors and body pain. This study indicated that age was not a risk factor of body pain intensity and interference in adulthood. A previous study showed that at higher levels of pain intensity, younger adults reported higher pain interference than their older counterparts [33]. The possible reason is that an age gap of two to three years between people may be insufficient to cause obvious differences in adults (45–53 years old). Similarly, previous research revealed that adults with low and high levels of pain intensity have no significant age slopes [33]. Further longitudinal studies are required to confirm the associations between age and body pain intensity and interference among the Chinese middle-aged population. This study also found that people who were married or cohabitation were less likely to have a higher trend of body pain intensity and interference than people who were unmarried/divorced or separated/widowed. A previous study showed that marriage was associated with pain-related symptoms [16]. This study further confirms this association between marital status and pain among middle-aged adults in China. In addition, this study found that females were more likely to have a higher body pain intensity rating. A study in the United States also reported greater rates of chronic widespread pain among women in the age groups of 40–64 years than males [14]. However, no unifying theory explains the gender difference of the response to pain intensity [34].

This study also found that people who had poor sleep quality, had poor relationships with their families, or were not satisfied with their current living conditions were associated with a greater likelihood of higher body pain intensity rating among middle-aged adults. A previous study further confirmed that people who had poor sleep quality were associated with a greater likelihood of higher body pain among middle-aged adults in the Chinese population [35]. Because the quality of life rating is subjective and related to a people’s life expectations [36], people who have poor relationships with their family, or are not satisfied with their current living conditions might increase their expectations for life. Another possible reason is that individuals who reported high levels of social support also reported greater pain [37]. Humans are primed to feel pain intensity and express attenuated vulnerability in more intimate social contexts [38]. Another explanation is that those people who have poor relationships with their families, or were not satisfied with their current living conditions, had immature coping styles and psychological symptoms for a long time to make sub-optimal decisions, and have low passion, especially when they encounter difficulties [19]. When one has a poor capacity to deal with unfavorable events, the probability of persistent physical pain increases [39]. Pain may be better differentiated when people are satisfied with their lives [40].

Participants with a lower socioeconomic position have higher body interference. This study found that people who had a low education level, were unemployed, and had low income were more likely to have higher body interference rating. Higher pain interference has been associated with greater psychopathology and poorer psychiatric treatment response [24,25]. Therefore, a possible implication of observation is that participants with a lower socioeconomic position are more likely to have greater psychopathology and poorer psychiatric treatment response. A previous study also showed that people who have a lower education level and are suffering from economic pressures were more likely to have higher pain interference [19]. However, people who are more than 56 years old with high education levels and income tend to have lower pain interference [41]. A previous study showed that the differing magnitudes of socioeconomic status might have important health implications when quantifying inequalities in life satisfaction and self-rated health [42], as well as positive attention and higher expectations of health. Self-perception of health status was related to pain [43] and participants with a lower socioeconomic position were likely to have self-perception. When a gap exists between expected and actual status, pain interference may improve [44]. A possible reason is the different characteristics between middle age and older people. According to the socioemotional selectivity theory, with the increase of age, people tend to add positive behaviors and reduce negative ones [45].

This study also found that people who had poor relationships with their family and were not satisfied with their current living conditions were more likely to have a higher body pain interference rating. A previous study showed that older women who are suffering from economic pressures and depressive mood were more likely to have a higher pain interference rating [19]. Similarly, a study showed that people, particularly women in their fifties and men in their thirties, showed higher pain interference when living with a family member [46]. These observations are broadly consistent with the “social signaling” perspective of human pain behaviors that are influenced by the social environment in which pain is expressed [47]. Moreover, given familial aggregation, a previous study showed a genetic relationship among chronic pain syndromes [48]. People who reported pain was associated with a greater likelihood of identifying other family members with pain than pain-free cohorts [29]. These findings help explain the causes behind pain interference in adulthood. Body pain interference is also can be prevented, and adopting an effective pain management improves health-related quality of life [7].

### Limitations

This study has several limitations that must be noted when considering its findings and contributions. First, this study applied the SF-36 scale, which might not be the best approach for assessing pain. Specifically, this scale contained only two items for evaluating pain. Given that this study focuses on body pain, this variable must be measured thoroughly. Future research may use the McGill Pain Questionnaire [28] as an alternative. Second, the cross-sectional survey data do not permit a reliable inference of causality. Third, the participants were relatively homogeneous in terms of race/ethnicity. Therefore, future investigations must adopt more heterogeneous samples to generate more generalizable results. Fourth, body pain intensity and interference in this study were correlated with socioeconomic status. Although several variables related to socioeconomic status (i.e., gender, age, educational level, and income) were carefully adjusted, residual confounding by socioeconomic status is still possible. Fifth, this study included only adults aged 45–53 years old; therefore, the findings may not apply to younger or older individuals.

## 5. Conclusions

An estimated 64.1% of middle-aged adults reported body pain, and 45.7% of middle-aged adults reported body pain interference. Respondents who were female, unmarried/divorced or separated/widowed, had a negative relationship with their family, had poor sleep quality and were not satisfied with their current living conditions had higher body pain intensity. Respondents who were unmarried/divorced or separated/widowed, with a low education level, were unemployed, had lower incomes, had a negative relationship with their family, and were not satisfied with their current living conditions had higher body pain interference. These findings provide possible interventions for improving body pain intensity and interference among middle-aged people. These factors should be taken into consideration in the pain management and prevention, which may help to relieve the stress of pain among middle-aged adults.

## Figures and Tables

**Table 1:** Characteristics of the study participants according to age group in adults (45–53 years old) in Chongqing, China (*n* = 1224, %).

Items	Total Population	Age Group		
	*n* = 1224	51–53 Years *n* = 299	48–50 Years *n* = 455	45–47 Years *n* = 470
Gender/male	56.1	55.9	56.7	55.7
Education level				
Basic education	15.3	18.7	17.6	10.9
Secondary education	49.8	54.2	50.1	46.8
Higher education	34.9	27.1	32.3	42.3
Marital status				
Unmarried/divorced or separated/widowed	7.76	8.03	8.57	6.81
Married or cohabitation	92.24	91.97	91.43	93.19
Job conditions				
Unemployed	44.0	36.8	47.7	44.9
Employed	45.8	43.5	41.8	51.1
Retire at home	10.2	19.7	10.6	4.0
Average monthly income				
<¥850	22.3	23.1	24.4	19.8
¥851–¥1,600	34.9	41.8	35.0	30.4
>¥1,601	42.8	35.1	40.7	49.8
Regular physical activity				
Seldom	27.7	26.1	25.7	30.6
Sometimes	49.3	50.5	50.3	47.7
Usually	23.0	23.4	24.0	21.7
Regular daily activities				
Seldom	37.0	41.5	35.8	35.3
Sometimes	56.5	54.2	57.4	57.0
Usually	6.5	4.4	6.8	7.7
Sleep quality				
Poor	8.7	8.0	9.5	8.3
Average	51.3	53.2	51.9	49.6
Good	40.0	38.8	38.7	42.1
Relationship with family				
Poor	1.8	1.3	2.0	1.9
Average	22.5	18.4	25.2	22.3
Harmony	75.7	80.3	72.8	75.7
Current living conditions				
Not satisfied	5.3	4.4	5.9	5.3
Average	42.0	37.8	43.3	43.4
Satisfied	52.7	57.9	50.8	51.3
Body pain intensity				
None	35.9	34.5	35.0	37.7
Very slight	28.8	29.8	30.6	26.6
Slight	25.5	24.8	25.7	25.7
Average	7.7	10.0	6.2	7.7
Severe	1.9	1.0	2.4	1.9
Extremely severe	0.3	0.0	0.2	0.4
Body pain interference				
Not at all	54.3	53.2	49.9	59.2
A little	34. 6	34.5	37.4	31.9
Moderate	9.9	11.7	10.8	7.9
Severe	1.1	0.7	1.5	1.1
Extremely	0.2	0.0	0.4	0.0

**Table 2:** Ordered Multivariate logistic regression analysis of body pain intensity in adults (45–53 years old) in Chongqing, China.

Parameter	Category	Model 1				Model 2			
		Estimate	SE	95% CI	*p*-Value	Estimate	SE	95% CI	*p*-Value
Intercept1		−3.326	0.733	−4.941, −1.995	<0.000	−3.335	0.737	−4.956, −1.996	<0.001
Intercept2		−1.138	0.494	−2.117, −0.176	0.021	−1.147	0.500	−2.137, −0.174	0.022
Intercept3		0.522	0.472	−0.408, 1.448	0.269	0.514	0.478	−0.427, 1.451	0.283
Intercept4		2.235	0.477	1.299, 3.173	<0.001	2.228	0.483	1.281, 3.178	<0.001
Intercept5		3.508	0.482	2.562, 4.456	<0.001	3.502	0.488	2.545, 4.461	<0.001
Age	51–53 years					0.120	0.141	−0.157, 0.396	0.395
	48–50 years					−0.037	0.123	−0.277, 0.203	0.764
	45–47 years (reference)								
Gender	Male	−0.318	0.110	−0.534, −0.102	0.004	−0.322	0.110	−0.539, −0.106	0.004
	Female (reference)								
Educational level	Secondary Education	−0.199	0.160	−0.513, 0.115	0.213	−0.193	0.160	−0.508, 0.121	0.228
	Higher Education	−0.081	0.193	−0.459, 0.297	0.674	−0.071	0.194	−0.450, 0.309	0.715
	Basic Education (reference)								
Job conditions	Employed	−0.036	0.118	−0.267, 0.194	0.757	−0.044	0.118	−0.275, 0.187	0.712
	Retire at home	−0.150	0.185	−0.513, 0.212	0.418	−0.181	0.188	−0.551, 0.186	0.334
	Unemployed (reference)								
Marital status	Married or cohabitation	−0.467	0.200	−0.861, −0.074	0.020	−0.471	0.200	−0.864, −0.079	0.019
	Unmarried/divorced or separated/widowed (reference)								
Average monthly income	¥851 to 1,600	−0.090	0.154	−0.392, 0.213	0.560	−0.094	0.154	−0.396, 0.209	0.542
	>¥1,601	−0.177	0.174	−0.518, 0.164	0.309	−0.175	0.174	−0.516, 0.167	0.316
	<¥850 (reference)								
Regular physical activity	Sometimes	−0.119	0.1277	−0.369, 0.131	0.350	−0.119	0.128	−0.369, 0.132	0.352
	Usually	−0.008	0.1555	−0.313, 0.297	0.958	−0.005	0.156	−0.310, 0.300	0.973
	Seldom (reference)								
Have a regular daily life	Sometimes	0.181	0.1182	−0.050, 0.413	0.125	0.188	0.118	−0.044, 0.421	0.112
	Usually	−0.072	0.2286	−0.522, 0.375	0.753	−0.055	0.229	−0.507, 0.394	0.811
	Seldom (reference)								
Sleep status	Average	−0.052	0.1979	−0.439, 0.337	0.794	−0.051	0.198	−0.439, 0.338	0.798
	Good	−0.485	0.2074	−0.892, −0.078	0.020	−0.479	0.208	−0.886, −0.072	0.021
	Poor (reference)								
Condition of getting along with family	Average	−0.788	0.4080	−1.590, 0.015	0.053	−0.783	0.407	−1.583, 0.018	0.055
	Harmony	−1.086	0.4056	−1.884, −0.289	0.007	−1.091	0.405	−1.887, −0.295	0.007
	Poor (reference)								
Current living conditions	Average	−0.664	0.2555	−1.167, −0.165	0.009	−0.670	0.256	−1.174, −0.170	0.009
	Satisfied	−1.023	0.2647	−1.545, −0.506	0.000	−1.036	0.265	−1.558, −0.518	<0.001
	Not satisfied (reference)								

CI: confidence interval; SE: standard error; Model 1 was not adjusted for age group; Model 2 was adjusted for age group.

**Table 3:** Ordered Multivariate logistic regression analysis of body pain interference in adults (45–53 years old) in Chongqing, China.

Parameter	Category	Model 1				Model 2			
		Estimate	SE	95% CI	*p*-Value	Estimate	SE	95% CI	*p*-Value
Intercept1		−2.922	0.841	−4.888, −1.448	0.000	−3.079	0.845	−5.049, −1.596	0.000
Intercept2		−0.825	0.520	−1.861, 0.182	0.113	−0.980	0.526	−2.027, 0.038	0.062
Intercept3		1.571	0.481	0.626, 2.515	0.001	1.420	0.487	0.463, 2.376	0.004
Intercept4		3.774	0.495	2.804, 4.748	<0.001	3.628	0.500	2.647, 4.613	<0.001
Age	51–53 years	-	-		-	0.202	0.156	−0.104, 0.5076	0.195
	48–50 years	-	-		-	0.266	0.136	−0.000, 0.5315	0.050
	45–47 years (reference)								
Gender	Male	−0.214	0.122	−0.452, 0.024	0.078	−0.218	0.122	−0.457, 0.020	0.073
	Female (reference)								
Educational level	Secondary Education	−0.674	0.166	−1.000, −0.348	<0.001	−0.655	0.167	−0.983, −0.328	<0.001
	Higher Education	−0.874	0.206	−1.278, −0.470	<0.001	−0.844	0.207	−1.251, −0.439	<0.001
	Basic Education (reference)								
Job conditions	Employed	−0.617	0.131	−0.875, −0.360	<0.001	−0.609	0.132	−0.868, −0.352	<0.001
	Retire at home	−0.289	0.200	−0.683, 0.100	0.148	−0.323	0.202	−0.723, 0.071	0.110
	Unemployed (reference)								
Marital status	Married or cohabitation	−0.517	0.219	−0.946,−0.087	0.018	−0.517	0.219	−0.945, −0.086	0.018
	Unmarried/divorced or separated/widowed (reference)								
Average monthly income	¥851 to 1,600	−0.722	0.165	−1.046,−0.399	<0.001	−0.721	0.165	−1.046, −0.398	<0.001
	Above ¥1,601	−0.784	0.188	−1.153,−0.417	<0.001	−0.768	0.188	−1.138, −0.401	<0.001
	Below ¥850 (reference)								
Regular physical activity	Sometimes	0.060	0.140	−0.213, 0.335	0.668	0.044	0.140	−0.230, 0.319	0.756
	Usually	−0.130	0.173	−0.469, 0.208	0.452	−0.145	0.173	−0.484, 0.194	0.403
	Seldom (reference)								
Have a regular daily life	Sometimes	0.139	0.130	−0.116, 0.395	0.288	0.137	0.131	−0.118, 0.394	0.293
	Usually	−0.038	0.252	−0.537, 0.453	0.880	−0.014	0.253	−0.514, 0.478	0.956
	Seldom (reference)								
Sleep status	Average	0.205	0.221	−0.224, 2.642	0.353	0.207	0.221	−0.223, 0.644	0.350
	Good	−0.281	0.232	−0.732, 0.178	0.225	−0.276	0.232	−0.727, 0.183	0.235
	Poor (reference)								
Condition of getting along with family	Average	−0.775	0.426	−1.613, 0.063	0.069	−0.786	0.427	−1.625, 0.054	0.066
	Harmony	−0.864	0.422	−1.694, −0.032	0.041	−0.871	0.423	−1.703, −0.038	0.040
	Poor (reference)								
Current living conditions	Average	−0.910	0.264	−1.429, −0.393	0.001	−0.918	0.264	−1.4387, −0.400	0.001
	Satisfied	−1.262	0.275	−1.802, −0.724	<0.001	−1.280	0.275	−1.821, −0.741	<0.001
	Not satisfied (reference)								

Model 1 was not adjusted for age group; Model 2 was adjusted for age group.
